# GnRH analogue followed by surgery in treatment of vaginal leiomyoma-a case report

**DOI:** 10.1097/MD.0000000000024911

**Published:** 2021-02-26

**Authors:** Yinxia Liu, Xiaoli Wang, Yuedong He

**Affiliations:** Department of Obstetrics and Gynecology, Key Laboratory of Birth Defects and Related Diseases of Women and Children, Ministry of Education, West China Second University Hospital, Sichuan University, Chengdu, People's Republic of China.

**Keywords:** GnRH analogue, surgery, vaginal leiomyoma

## Abstract

**Rationale:**

Vaginal leiomyoma is a rare type of leiomyoma that occurs on the wall of vagina. Treatment for vaginal leiomyoma is varied and is based on the location and size of the leiomyoma.

**Patient concerns:**

In this case, a 24-year-old newly married Chinese woman complained of dyspareunia. The physical examination revealed a solid mass on the anterior wall of vagina. It almost filled up the whole vagina cavity.

**Diagnosis:**

Transvaginal ultrasound showed a tumor on the anterior wall of vagina. Pelvic computed tomography (CT) and magnetic resonance imaging (MRI) also confirmed the tumor on vaginal wall. Fine needle aspiration biopsy confirmed fibrous and smooth muscle tissue in the tumor, and immunohistochemical examination found the estrogen receptor (ER) and progesterone receptor (PR) were positive.

**Interventions:**

6 courses of gonadotropin-releasing hormone (GnRH) analogue were given before the patient underwent complete surgical resection through vagina.

**Outcome:**

No postoperative complications occurred, and the patient was discharged from the hospital 3 days after surgery. Follow-up after 3 months revealed negative symptoms of genitourinary system. No sign of recurrence was found.

**Conclusion:**

In this case, vaginal leiomyoma was diagnosed with help of imagological examinations like ultrasound, CT, and MRI, as well as pathological examination like fine needle aspiration biopsy. Preoperative GnRH analogue treatment can ensure smooth surgical procedure, and reduce blood loss during surgery.

## 1 Introduction

Leiomyoma is a common gynecological benign tumor among childbearing women.^[1]^ Corpus uteri, cervix, broad ligament, vagina, paraurethra are sites where leiomyoma may occur. Vaginal leiomyoma is a rare type leiomyoma. Clinical symptoms like dysuria, urinary frequency and urgency, retention of urine, dyspareunia may appear because the vaginal leiomyoma is located close to the urethral tract.^[2]^ Besides, treatments for vaginal leiomyoma are varied based on the location and size of the mass.^[3,4]^ Here, we present a case of vaginal leiomyoma and its treatment.

## 2 Case report

This case report was approved by the patient with written informed consent for publication.

A 24-year-old female, gravida 0, para 0, presented to the gynecology Department of a Chinese Women and Children Hospital, complaining of dyspareunia for 6 months. The patient had no sexual intercourse before marriage. Six months ago, she got married, but the new couple experienced dyspareunia. She did not have urinary frequency or urgency, or other symptoms associated with urinary tract. Her menstrual history was normal.

**Figure 1:**
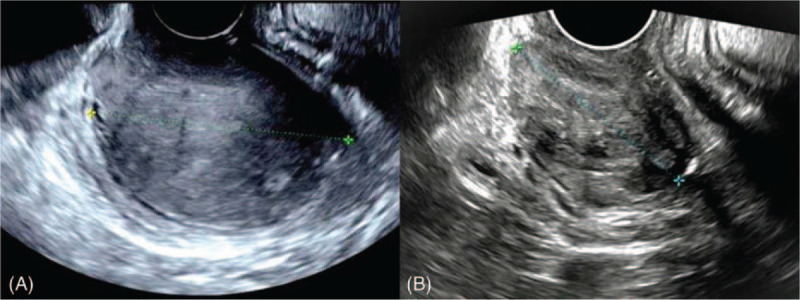
Transvaginal ultrasound showed a hypo-echoic mass on the anterior vaginal wall before (A) and after (B) treatment of GnRH analogue.

**Figure 2:**
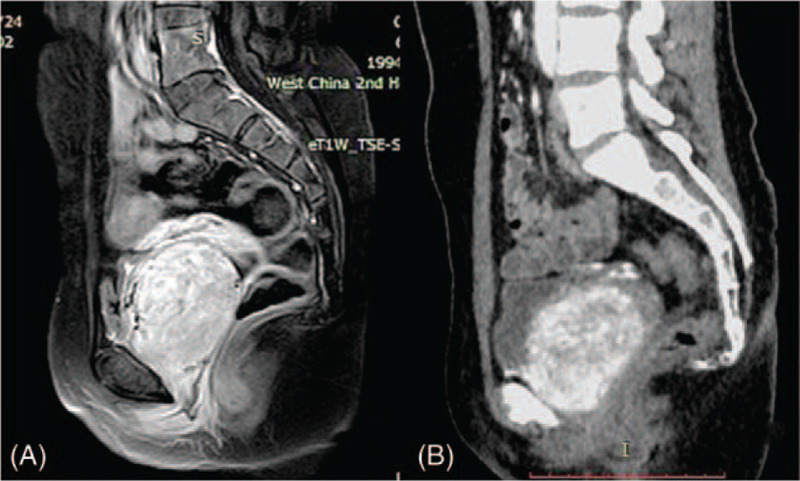
MRI (A) and CT (B) showed a mass on the anterior vaginal wall.

**Figure 3:**
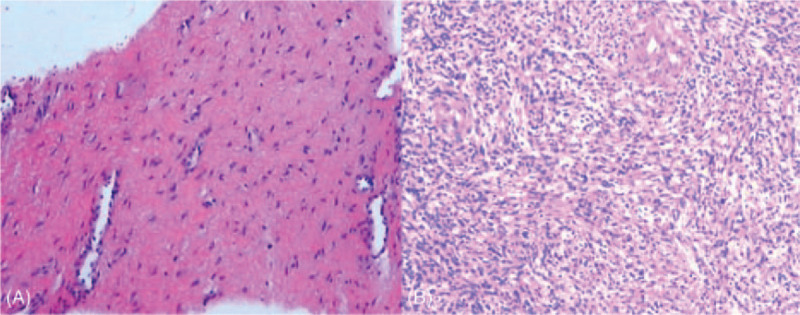
Presurgical biopsy found fibrous and smooth muscle tissue in the tumor (A, 400×), and postsurgical specimen confirmed it to be spindle cell tumor (B, 200×).

Speculum examination can only visualize the lower third part of the vagina, and a solid mass was seen extruding from middle part of anterior vaginal wall and it almost filled the whole vaginal cavity. Bimanual examination revealed a round, hard, fixed mass on the middle third of anterior vaginal wall, and upper third part of vagina, as well as cervix, cannot be detected. Transvaginal ultrasound revealed a 5.7 × 5.2 × 6.6 cm hypo-echoic mass on the anterior vaginal wall, with well-defined margin (Fig. 1-a). Uterus and adnexa were unremarkable. Pelvic MRI also revealed the mass on anterior vaginal wall. It was isointense to muscle on T1-weighted imaging and slight hyperintense to muscle on T2-weighted imaging. The signal was enhanced after gadolinium injection (Fig. 2-a). The mass was also enhanced on computed tomography (CT) examination on the enhanced scanning (Fig. 2-b). Fine needle aspiration biopsy of the mass revealed fibrous and smooth muscle tissue (Fig. 3-a), and immunohistochemical examination found the estrogen receptor (ER) and progesterone receptor (PR) were positive.

The tumor filled almost the whole vaginal cavity, and upper third part of vagina, and the cervix could not be detected. Also, biopsy revealed benign smooth muscle tissue with positive ER and PR. 3.75 mg Triptorelin, a kind of GnRH agonist, was given to the patient monthly through subcutaneous injection for 6 months before surgical excision. The patient suffered from menopausal symptoms after injection of GnRH agonist and Remifemin, a natural formula which helps to reduce the unpleasant physical and emotional symptoms associated with menopause, and calcium tablets were added during the treatment. The bimanual examination found the tumor shrank obviously after treatment of GnRH analogue and ultrasound showed the tumor decreased to 4.2 × 2.3 × 3.2 cm (Fig. 1-b).

Surgery was done via vagina approach. Before surgery, a catheter was indwelled to indicate the urethra. Midline incision was applied on the anterior wall of vagina. A 4 cm in diameter, fixed, hard, grey tumor which was encapsulated was found after separating the surrounding tissue carefully. The tumor was not associated with urethra. It was removed completely and sent for pathological examination. Total blood loss during surgery was about 30 ml. The pathologic examination confirmed it to be benign spindle cell tumor with abundant small vessels, diagnosed as leiomyoma (Fig. 3-b).

Urethra catheter was removed 48 hours after surgery and the patient was discharged from hospital 72 hours after the surgery. No discomfort was observed during the hospital stay.

The patient came back for a physical examination 3 months later. The wound had healed well, and bimanual examination revealed a soft anterior vaginal wall.

## 3 Discussion

In this case report, we presented the diagnosis and treatment of a case of vaginal leiomyoma. Preoperative GnRH analogue significantly reduced tumor size and made the surgical resection easier and safer.

Leiomyoma are found in up to 70% of women of reproductive age.^[1]^ Vaginal leiomyoma is a rare type of leiomyoma. Liu calculated in 1988 that about 250 cases of vaginal leiomyoma had been reported.^[5]^ Many cases had been reported since then. According to the reports, vaginal leiomyoma most commonly affects women at 35 to 50 years old.^[2]^

Vaginal leiomyoma can grow anywhere in vagina, but they are more commonly found in the anterior wall.^[6–9]^ Vaginal leiomyoma can expand from vaginal wall with broad base, it can also attach to vaginal wall with a small peduncle. Normally, vaginal leiomyoma is a benign tumor, however, vaginal leiomyosarcoma are also reported.^[10–12]^ Vaginal leiomyoma can also occur several years later after hysterectomy.^[3,13]^

Symptoms of vaginal leiomyoma varies from asymptomatic to excessive discharge, abnormal uterus bleeding, urinary frequency and urgency, urine retention, dyspareunia, and so on, based on the location and size of the leiomyoma.^[2]^ Gowri et al reported a huge vaginal leiomyoma presenting as a gluteal swelling with pus discharging per vagina.^[4]^ Liu reported that symptoms appear when the vaginal leiomyoma reached 6 cm or larger.^[5]^ Ultrasound, pelvic MRI and CT are valuable in diagnosis of vaginal leiomyoma. Typical appearance of leiomyoma in ultrasound, MRI and CT are described in several reports.^[14]^ However the golden standard for diagnosis is tissue biopsy.

Local excision is the common treatment of vaginal leiomyoma.^[3]^ In most cases, the leiomyoma is resected directly. However, adjuvant treatments are also applied to help surgical resection. Bapuraj et al reported the first case of huge vaginal leiomyoma treated with embolization before surgery in case of uncontrolled bleeding during surgery.^[15]^ In the case we presented, GnRH analogue was applied before surgery to decrease tumor size, because ER and PR were positive in the biopsy tissue. GnRH analogue influences the function of ER and PR by influencing function of hypothalamo-pituitary-gonadal axis,^[16]^ and preoperative GnRH analogue was confirmed to reduce fibroid volume in a meta-analysis of randomized controlled trials.^[17]^ Menopause symptoms are common side effect of GnRH analogue. Hormone add-back therapy are confirmed to attenuate hypoestrogenic effects.^[18]^ Natural formula like Remifemin can also help to reduce unpleasant physical and emotional symptoms associated with menopause.^[19,20]^

According to the location and size of leiomyoma, the surgeries were done via vaginal approach or abdominoperineal approach as reported. In most cases, vaginal leiomyoma was resected via vaginal approach. However, abdominoperineal approach was also used in huge vaginal leiomyoma, and Gowri et al reported a case of huge vaginal leiomyoma excised through abdominoperineal approach.^[4]^ Recurrence of vaginal leiomyoma is also reported. Dhaliwal et al reported a case of recurrent huge vaginal leiomyoma 5 years after primary vaginal leiomyoma, and excision of ovaries was suggested if the patient had intact ovarian function when recurrence developed.^[21]^

In conclusion, application of GnRH analogue before surgery helps to decrease the volume of vaginal leiomyoma, ease the surgical procedure, and reduce blood loss during surgery.

## Acknowledgments

The authors would like to thank the colleagues in pathologic department and radiologic department for providing the pictures in this case. The authors also would like to thank the patient for agreeing to reveal the case details for publication.

## Author contributions

**Conceptualization:** Yinxia Liu.

**Investigation:** Yinxia Liu.

**Methodology:** Yinxia Liu.

**Resources:** Yinxia Liu, Xiaoli Wang.

**Supervision:** Xiaoli Wang, Yuedong He.

**Validation:** Yinxia Liu.

**Visualization:** Xiaoli Wang.

**Writing – original draft:** Yinxia Liu.

**Writing – review & editing:** Xiaoli Wang, Yuedong He.
